# Skin Reactions to Pine Processionary Caterpillar *Thaumetopoea pityocampa* Schiff

**DOI:** 10.1155/2013/867431

**Published:** 2013-05-27

**Authors:** Domenico Bonamonte, Caterina Foti, Michelangelo Vestita, Gianni Angelini

**Affiliations:** Department of Biomedical Sciences and Human Oncology, Section of Dermatology, University of Bari, Piazza Giulio Cesare 1, 70124 Bari, Italy

## Abstract

Pine caterpillar, *Thaumetopoea pityocampa* Schiff, is a phyto- and xylophagous lepidopteran, responsible for the delay in the growth or the death of various types of pines. Besides nature damage, pine caterpillar causes dermatological reactions in humans by contact with its irritating larvae hairs. Although the dermatitis occurs among outdoor professionals, it is primarily extraprofessional. Contamination generally occurs in pinewoods, rarely in cities. Means of contamination comprise direct contact with the nest or the processional caterpillar and indirect contact with air dispersed hairs. The dermatitis is generally observed in late spring and particularly from April to June, among campers and tourers. The eruption has its onset 1–12 hours after contact with the hairs and presents with intense and continuous itching. Morphologically, it is strophulus-like and consists of papulous, excoriated, and pinkish lesions on an oedematous base. Diagnosis is usually straightforward. The pathogenetic mechanism of the affection is mechanical, pharmacological, and allergic in nature. Besides skin, *T. pityocampa* Schiff can involve the eyes and rarely the airways. Despite the considerable damages to humans and nature, pine caterpillar infestation is an underestimated problem; medical literature lists few studies, and often relevant information is referred to local media and popular wisdom.

## 1. Introduction

Among Mediterranean countries, on coastal regions, each year pines are assaulted by an apparently inoffensive insect: the pine caterpillar *Thaumetopoea pityocampa* Schiff. As a matter of fact, this caterpillar is strictly phyto- and xylophagous and thus survives by eating pine structures, destroying their branches and delaying their growth. Old pines are especially infested by a massive quantity of parasites and can die ensuing the invasion. The disruptive effects of pine caterpillar extend to man and pet animals, leading to various pathological conditions. Pine caterpillar hairs have been known since ancient times to adverse reactions, which do not confine to the skin but also involve the ophthalmic and respiratory systems.

The first clinical and pathogenetic descriptions on pine caterpillar were given by entomologists [1–3]. Many French authors followed the problem being widespread in certain west and south areas of France [4, 5]. In Italy, the Apulia region is particularly burdened by such environmental and medical matter, which is sometimes referred to by the media as a proper “nightmare” [6–10]. As of today, pine processionary is expanding northwards as a direct effect of global warming, which permits better survival of its larvae, in areas in which it would otherwise be unable to develop [11]. Despite the entity of the problem, the international literature reports only 2 studies concerning the prevalence of pine processionary cutaneous reactions: one in children [12] and one in the general adult population [13], while the largest available case series encompass 30 patients diagnosed with occupational immunologic urticaria from pine caterpillar [14].

In the present paper, besides clinical data, we report the main features of the biology and the geographical distribution of pine caterpillar, *Thaumetopoea pityocampa* Schiff.

## 2. Erucism and Lepidopterism 

Often used as synonyms, the 2 terms are not interchangeable. The first, erucism (from the Latin *eruca*: caterpillar), is peculiar to cutaneous pathology from caterpillars. Lepidopterism (from the Greek *lepís*: scale and *ptèron*: wing) is instead referred to pathology from butterflies. Pine caterpillar is not the only urticarial species.  Table 1 reports the most common Lepidoptera families, each grouping various species of urticarial caterpillars [15, 16]. To the Thaumetopoeidae family belong 3 urticarial species:

1. *T. pityocampa* Schiff, pine caterpillar,
2. *T. processionea* L., oak caterpillar,
3. *T. pinivora* Tr., Nord Europe pine caterpillar.

While the biological cycle of oak caterpillar differs from the pine species (larval life is considerably shorter in the former), the induced clinical symptoms are undistinguishable. Among Lepidoptera, genus *Hylesia* moths (of the Saturniidae family) are also equipped with urticarial hairs, which are responsible for the “papillonite Guyane” [17], also named “Caripito itch” (from an epidemic form that broke out in Caripito docks in Venezuela) [18].

### 2.1. *Thaumetopoea pityocampa* Schiff

**Figure 1:**
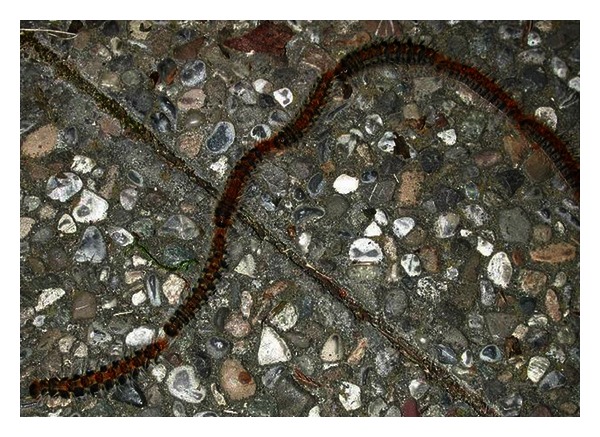
*Thaumetopoea pityocampa* Schiff larvae in a procession fashion.

*T. pityocampa* Schiff, or pine caterpillar, is a “phenomenal” insect. The term comes from the Greek *cámpa* (caterpillar), *pítys* (pine), *poieo* (does), *tháuma* (wonders).  Table 2 shows its classification. The biological cycle encompasses 2 phases: an aerial as well as a ground one [4]. The former begins with the moth formation and includes the evolution from eggs to larvae. Female moths, once fecundated, lay eggs (70–300) only once at the extremities of pine branches. Larvae hatch from eggs in a 5-6-week timeframe. Showing a gregarious behavior during the larval phase, caterpillars stay together and attached to pine needles. While devouring the latter, they weave a net creating “tent” nests, typically placed on tree tops. Caterpillars move among branches and also among trees in order to feed. These movements happen in a procession fashion (nose to tail columns), usually at night (Figure 1).

During the aerial phase, the pine processionary evolves through 5 instar stages (L1, L2, L3, L4, L5). Climatic conditions, warm weather in particular, are essential to larvae development. Pine caterpillar does not tolerate temperature above 25°C or below 5°C, the optimal range being 20–25°C. Aerial larval phase ends between March and June. At this time caterpillars look for a feasible ground to infiltrate, in a warm and well-lighted area, beginning the ground phase. The transformation in chrysalis thus occurs. The following turn from chrysalis to moth takes a month. The adult retains the same name (*T. pityocampa*), and it is a nocturnal moth, generally flying around light sources.

Pine processionary cycle is therefore annual. Based on climatic conditions, it can span among years (2–5). Even the above 2 biological phases can vary in duration. For these very reasons, human pathology from pine caterpillar can be observed all year round.

For protective purposes, processionary larvae have developed an urticarial apparatus. At the fourth and fifth instar stages, their tegument comprises two different kinds of hairs: true non-removable hairs and removable urticarial setae, disposed dorsally and medially on the first 8 abdominal larva segments, thus sparing the last caudal two. The setae, displaced on “mirror-like” morphology apparatus, are laid out on the segments of 4 articular larva scales with a density of 60.000/mm^2^ circa, or rather 120.000 for each “mirror” and 1 million for each caterpillar [13]. Furthermore, they vary in length from 100 to 250 *μ*m and present pointed spikes towards the distal end and a proximal extremity normally infixed in cuticular pads.

Urticarial hairs penetrate through human skin by means of the proximal extremity. Typically, these hairs do not show any superficial holes but are hollow for most of their axis. They have defensive action and are expelled in great quantities when the caterpillar is somehow menaced, through the contraction of intersegmental muscles. Given the dimensions, such hairs are invisible; thousands are projected in the air as a fine powder.

### 2.2. Pathogenic Effects of Pine Caterpillar

Pine processionary is common along the whole Mediterranean coast and in France, Italy, Israel, and Lebanon in particular. It affects every species of pines and Cedrus trees, with a marked preference for black pines.  Table 3 lists the most frequently infested pines. It is of valuable consideration that pine processionary infestations in forests can lead to disastrous outcomes, both in terms of environment and economy. In ancient times some Latin authors had already reported the phenomenon. Rome passed a specific law against concoctions containing pine processionary, among other ingredients, administered in order to break magical spells [4].

The pathogenic effects of pine processionary are not limited to the skin but extend to the eyes and, more rarely, to the respiratory system. The dual pathogenic mechanism is as follows:

1. direct contact with nests or caterpillars is the cause of the processionary dermatitis;
2. aeromediated contact with air dispersed urticarial hairs is the cause of the skin, as well as the ocular and the respiratory affections.

Contamination is common in pine forests (70% of cases), less frequent outside forests (26.8%), and exceptional in urban environment [6, 7].

Aeromediated contact forms are the most commonly observed. The greatest part takes place from March to June, with a peak in April and May; obviously this may differ in relation to weather and caterpillar biological cycle variations.

## 3. Processionary Dermatitis

Processionary dermatitis is observed in occupational settings (lumberjacks, woodcutters, other forestry personnel, residential gardeners, nurserymen, stockbreeders, resin collectors, and entomologists) and even more in extraoccupational situations, such as tourers and campers. Individuals of every age can be affected, especially children who tend to play with these larvae [19–21].

Aeromediated contamination is favored by the wind; sweating also eases dermatitis onset. Eruption severity and distribution depend on exposition modality and intensity. Face, neck, forearms, interdigital spaces, and hands dorsum are the most involved body areas. Based on contact modality, lesions can be confined (direct contact) or rather multiple and extended (aeromediated contact), given that irritant hairs can pass through clothes. The eruption onset dates 1–12 hours from contact, or rarely, days after. 

**Figure 2:**
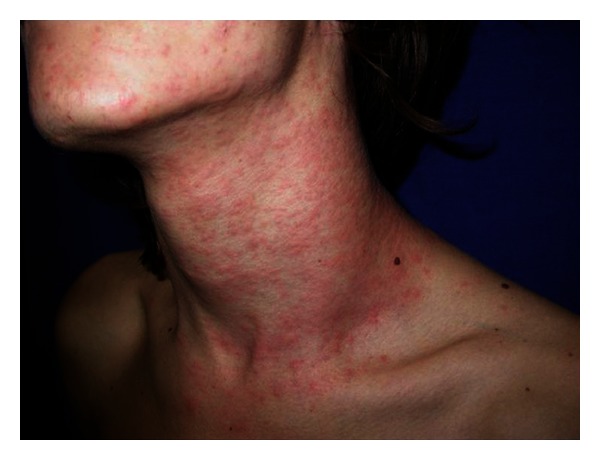
Aeromediated contact papulo-urticarial eruption due to setae of pine caterpillar.

**Figure 3:**
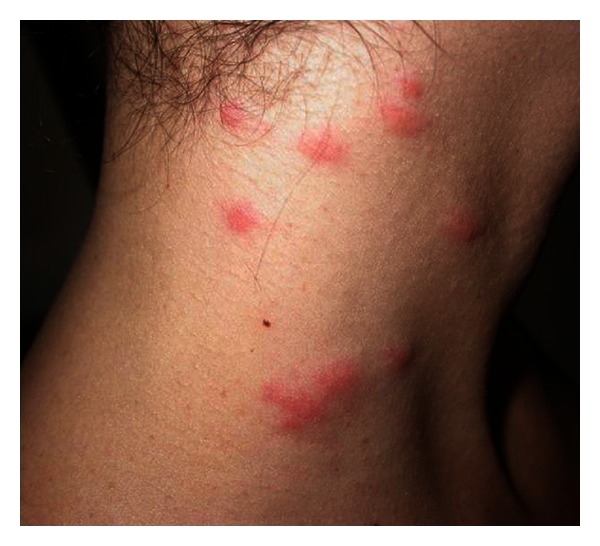
Strophulus-like lesions due to setae of pine caterpillar.

Itching is intense and continuous, with intermitting worsening. Clinically, the eruption manifests with rose to bright red, round macules and papules, of 3–8 mm in diameter, overlapping an urticarial base (Figure 2). Papules can be surmounted by vesicles [7]. Purpuric and scratching lesions are common findings. Oftentimes clinical characteristics mimic those of strophulus (Figure 3), sometimes with bullous lesions. At the eyelids the eruption can become evident with a more or less conspicuous edema. Linear and figurated papulourticarial lesions are seen in children who let caterpillars stroll on the skin. Although rarely, skin manifestations can parallel systemic symptoms, such as malaise, fever, and anaphylaxis syndrome [16, 22]. The incidence of the latter has been shown to be as high as 40% in a specific case series [14]. Cutaneous lesions evolve in 3-4 days and leave a brownish macule which later resolves in 1-2 weeks. An atypical case has been reported in the Italian literature and cited in the international: a farmer who had developed an ulcerative dermatitis of the penis after he had manipulated pine processionary nests (*Cnethocampa pinivora*) and had afterwards masturbated [23, 24].

### 3.1. Pathogenetic Mechanisms

The mechanism is dual, mechanic (skin infixion by hairs), and pharmacological [13, 16, 20, 21, 24]. The latter has been demonstrated in 1907 when Tyzzer, exposing erythrocytes to larvae hairs, noticed spherocytes formation, indicating the presence of toxic substances in the hairs [25]. The pathogenesis of processionary dermatitis from the *Euproctis chrysorrhoea* Linn moth has been studied by De Jong and Bleumink [26, 27]. It is likely that the same mechanisms are valid for other processionary species, although hair venom composition in the various Lepidoptera families is yet to be completely recognized. Shared venom components include histamine, histamine releasers, serotonin, and proteases [28, 29]. In 1986, Lamy and Coll isolated a protein, thaumetopoein, from pine processionary hairs [30]. This protein directly acts on mastocytes, inducing degranulation, validating a nonspecific urticarial effect of such caterpillars.

However, besides the direct histaminergic mechanism, reactions to *T. pityocampa* have long been suspected to be associated to IgE-mediated hypersensitivity [31]. As a matter of fact, recently published studies have demonstrated through *in vitro* and *in vivo* tests that an IgE-mediated mechanism is involved in most cases by *T. pityocampa* in adults [19, 32] and that the allergenic potency dramatically increases during larvae development, peaking at the L5 instar stage [33]. In particular, a 2012 study showed that setae contain a complex mixture of at least 70 proteins, including 7 allergens which are delivered to the skin by penetration of the setae [34]. The latter comprises minute amounts of proteins enclosed in a chitin-based envelope. Chitin exposure has been shown to induce expression of interleukin (IL)-4 and IL-13 and thus of eosinophils and basophils. Therefore, it has been proposed that exposure to chitin might be the primary trigger in allergy development [35]. Additionally, data show that *T. pinivora* setae are able to penetrate the outer skin layer and remain therein for up to 3 weeks, potentially releasing allergens that could trigger and/or enhance an immune allergic reaction in the host [36].

### 3.2. Diagnosis

Diagnosis of pine processionary dermatitis, in both direct contact and aeromediated forms, is generally straightforward. History of residing, passing through or nearby pine forests is of prime importance, as is the history of direct contact with caterpillars, the presence of strophulus-like lesions, the disposition of the latter, and the occurrence of the dermatitis in patient friends and family. Lesions stripping with tape and subsequent microscopic examination can demonstrate caterpillar hairs presence [37].

Histopathological studies on spontaneous lesions from processionary hairs are scarce [38]. Focal disruption of the stratum corneum, along with epidermis cells lysis and consequent intraepidermic vesicles, has been described in experimentally induced lesions. Hair fragments are usually visible. Perilesional skin appears spongiotic, while edema and a perivascular lymphocyte, neutrophil, and eosinophil infiltrate are apparent in the dermis. In a later stage the same features become more discernible, with intense spongiosis and intraepidermic bullae formation; in the dermis the infiltrate extends to the hypodermis and becomes lymphohistiocytic in composition [24, 38].

### 3.3. Cutaneous and Laboratory Testing

Patch tests with ether, alcohol, and saline filtrates result negative. On the other hand, prick tests with grinded hair filtrate turn positive with a variably marked urticarial reaction. These tests support the histaminergic urticarial activity of the substances, the necessity of skin scarification for the reaction to take place, as well as the need for hairs crushing in order to release the pathogenic substances.

*In vitro* test (IgE-immunoblotting) can be performed in patients with a positive prick test to confirm the allergic nature of the cutaneous reaction [34].

### 3.4. Therapy

Treatment is mainly supportive and shows scarce efficacy. Systemic antihistamines do not reveal great usefulness. Nevertheless their use is advised for. Topical steroids can accelerate lesions resolution, while systemic steroids are exceptionally utilized in severe cases. Topical anti-itching products containing menthol or phenol can be helpful in relieving pruritus. The usefulness of topical potassium dobesilate 5% cream has been recently reported [39].

## 4. Ocular Involvement

In approximately 10% of cases, cutaneous lesions associate with early or late ocular involvement, which can be tricky to diagnose correctly [5, 13]. Early ocular lesions are represented by immediate burning sensation, almost invariably unilateral, with hyperemia and edema of conjunctiva and eyelids. History discloses a mild trauma. The inflammatory reaction worsens over the following days, with photophobia, profuse tearing, and formation of conjunctival yellowish nodules. These nodules, which generally subsume caterpillar hair, gave name to the affection, known as ophthalmia nodosa.

Late ocular lesions are the consequence of hairs penetration inside the ocular globe. In the occurrence of hairs migration towards the inner structures, sclera involvement, iris nodules, glaucoma, keratitis, uveitis, cataract, and panophthalmitis can be observed [5, 12, 13, 19, 32].

## 5. Respiratory Involvement 

Respiratory involvement is rare and only anecdotally associated to pine processionary hairs inhalation. The upper airways are generally affected with rhinitis, cough, dysphagia, and dyspnea as a result of laryngeal mucosa direct irritation. Asthma crisis, thoracic pain, and risk of asphyxia are possible and rarely occur and require urgent treatment [5, 12, 13, 19, 32].

## 6. Conclusions

Medical literature lists a scarce number of observations and studies regarding pathology from pine processionary. In contrast, vast European coastal areas are burdened by this matter, often victim of both environmental and economic damages of considerable proportion, not to mention the ongoing expansion of the phenomenon towards northern previously unaffected areas due to global warming. In front of this, education on the subject is frequently demanded on inconsistent means such as local press and popular wisdom. Further investigation of the problem, both epidemiologically and pathogenetically, is therefore highly advisable.

## Figures and Tables

**Table 1:** Common urticarial Lepidoptera [15, 16]*.

Superfamily	Family	Species
Bombycoidea	Saturniidae	*Hylesia* sp.
	Lasiocampidae	*Dendrolimus punctatus *

Noctuoidea	Arctiidae	*Hyphantria cunea *
		*Euproctis chrysorrhoea *
	Lymantriidae	*E. edwardsi *
		*E. similis *

Zygaenoidea	Megalopygidae	*Megalopyge opercularis *
	Colchlididae	*Sibine stimulea *

Notodontidae	Thaumetopoeidae	*T. pityocampa* Schiff *T. pinivora* Tr. *Thaumetopoea processionea* L.

*The urticarial agents are processionary caterpillars among Thaumetopoeidae moths among other families.

**Table 2:** *Thaumetopoea pityocampa* Schiff.

Superorder	Mecopteroide
Order	Lepidoptera
Superfamily	Notodontidae
Family	Thaumetopoeidae

**Table 3:** Pines and cedars most commonly infested by *Thaumetopoea pityocampa *Schiff.

*Pines *
Austrian black pine
Corsican pine (*Larix decidua, L. europaea*)
Maritime pine (*Pinus pinaster, P. maritima*)
Sylvester pine (*Pinus sylvestris* L.)
Aleppo pine (*Pinus halepensis* Miller)
*Cedars *
Lebanon cedar (*Cedrus libani*)
Atlas cedar (*Cedrus atlantica*)
Cyprus cedar (*Cedrus brevifolia*)
